# Effect of advanced maternal age on ischemic stroke vulnerability in aged rats: Investigating on blood-brain barrier permeability and gene expression

**DOI:** 10.1016/j.nbas.2024.100125

**Published:** 2024-09-11

**Authors:** Samira Khayat, Hamed Fanaei

**Affiliations:** a Pregnancy Health Research Center, Zahedan University of Medical Sciences, Zahedan, Iran; b Department of Midwifery, School of Nursing and Midwifery, Zahedan University of Medical Sciences, Zahedan, Iran; c Department of Physiology, School of Medicine, Zahedan University of Medical Sciences, Zahedan, Iran

**Keywords:** Advanced maternal age, Aging, Stroke, MMP9, Occluding, VEGF

## Abstract

**Background:**

Advanced maternal age (AMA), commonly defined as pregnancy at or above 35 years old. Based on the evidence, this trend has raised concerns about potential health consequences for mothers, particularly in relation to ischemic stroke. Studies suggest that AMA may be associated with a higher risk of ischemic stroke in women due to physiological changes that impact vascular health and increase cardiovascular risk factors. The aim of this study was to investigate the effect of AMA on the extent of damage after ischemic stroke in aged rats.

**Methods:**

Female rats that gave birth at an old age (10 months) and at a young age (4 months) were subjected to ischemic stroke in old age (20 months) and subsequently compared.

We assessed neurological deficits, infarct volume, blood–brain barrier (BBB) permeability, TNF-alpha levels, total oxidant capacity, and gene expressions that play a role in BBB integrity (VEGF, Occludin, and MMP-9) following ischemic stroke.

**Results:**

There were significantly elevated levels of MMP-9 expression and reduced levels of occludin in AMA rats. Additionally, AMA rats had significantly higher levels of TNF-alpha and total oxidant capacity after experiencing an ischemic stroke. AMA rats showed significantly higher brain water content (BBB permeability), infarct volume, and neurological deficits compared to young-aged pregnancies.

**Discussion:**

Complex relationship between pregnancy-related physiological changes, aging, vascular gene expression, and inflammatory factors may play a role in the increased vulnerability observed in older pregnant rats. The similarities between pregnancy-related alterations and aging highlight the influence of advanced maternal age on susceptibility to ischemic stroke.

## Introduction

Advanced maternal age (AMA), commonly defined as pregnancy at or above 35 years old, has emerged as a prominent trend in contemporary society, driven by various socio-cultural and biological factors [1], [2]. The decision to delay childbearing, pursue career aspirations, and advancements in fertility treatments have contributed to a shift in reproductive patterns, leading to an increasing number of women opting for pregnancy at an older age [1], [2]. This trend has sparked concerns regarding the potential health implications for both mothers and offspring, particularly in relation to neurological outcomes [1], [2].

Stroke, a major cause of morbidity and mortality worldwide, represents a significant health concern that may be influenced by maternal age [3], [4], [5]. Previous studies have suggested a possible association between AMA and an elevated risk of stroke in women [3], [4], [5], yet the underlying mechanisms remain poorly understood.

Studies with human subjects have shown that AMA has notable effects on vascular health [3], [4], [5], [6], [7]. AMA is linked to a greater occurrence of risk factors like hypertension, diabetes, and obesity, all of which are recognized as factors that increase the risk of stroke [3], [5], [6], [7]. The aging process results in vascular alterations that can make individuals more susceptible to conditions such as atherosclerosis, thrombosis, and reduced vascular flexibility, all of which heighten the likelihood of experiencing a stroke [8]. Furthermore, AMA is linked to alterations in hormonal profiles. Specifically, AMA is associated with decreased peak gestational estradiol levels, which are essential for cardiovascular health protection [9], [10]. A cohort study that compared young mothers with AMA mothers revealed significantly reduced estradiol levels in the third trimester for AMA mothers [9]. The decline in estrogen levels during and after pregnancy at an advanced age may disrupt vascular function and elevate the risk of stroke [10]. Additionally, women of AMA may have a higher burden of comorbidities and preexisting conditions that synergistically heighten the risk of stroke [7], [10]. The combined impact of age-related changes, hormonal fluctuations, and preexisting health conditions can significantly increase the risk of stroke in older women who have experienced pregnancy at an advanced age [3], [7], [10]. It is crucial to grasp the intricate connection between Advanced Maternal Age (AMA), hormonal changes, vascular adjustments, and risk factors to comprehend the elevated stroke risk in these women [3], [6], [7]. These factors synergistically disrupt vascular equilibrium, promote a prothrombotic state, and impede cerebral blood flow, ultimately heightening the susceptibility to stroke in older women who have had pregnancies later in life [3], [7], [10].

Exploring the influence of AMA pregnancies on stroke-induced damage in older age could offer valuable insights for potential interventions to enhance outcomes for women with AMA. To bridge this knowledge gap, the current study was designed to examine the effects of AMA pregnancies on stroke-related damage in aging using a rat model. This study aimed to examine the effects of advanced maternal age on blood–brain barrier permeability, infarct volume, and neurological damage after stroke in old age using a rat model. We also compared gene expressions of VEGF, MMP9, and occluding, which play crucial roles in maintaining BBB integrity, as well as TNF-alpha and oxidative stress levels between rats that experienced pregnancy at an older age and those that were younger.

## Material and Methods

The Institutional Animal Research Ethics Committee of Zahedan University of Medical Sciences approved this study (ethical code: IR.ZAUMS.REC.1393. 6852). All chemicals used were sourced from Sigma-Aldrich (St. Louis, Missouri, US), unless specified otherwise.

### Animals

**Fig. 1:**
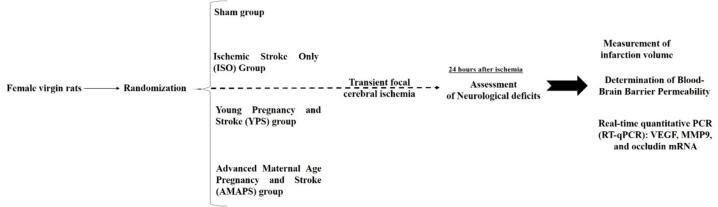
Experimental design overview.

Female Sprague Dawley rats were used for the experiments. The rats were procured from the Laboratory Animal Research Center at Zahedan University of Medical Sciences at 12 weeks old and assigned to either the young or aged group (Fig. 1).

The young rats conceived at 4 months of age, whereas the older rats (considered as advanced maternal age rats) conceived at 10 months old [11]. In human terms, this age for the older rats is equivalent to approximately 35 years old, which aligns with the concept of advanced maternal age in humans, taking into account developmental milestones like weaning, sexual maturity, skeletal maturity, and reproductive senescence [2], [11], [12].

The animals were housed in a vivarium with controlled room temperature and humidity, following a 12-hour light–dark cycle. They had free access to water and were provided with standard laboratory chow.

A total of 64 rats were randomly assigned to four groups (n = 16 per group), as follows:

1. Sham group: Rats in this group underwent sham procedures without experiencing pregnancy or stroke
2. Ischemic Stroke Only (ISO) Group: Rats did not experience pregnancy; however, at 20 months of age, the animals underwent stroke surgery.
3. Young Pregnancy and Stroke (YPS) Group: Rats experienced a pregnancy at 4 months of age, and at 20 months of age, the animals underwent stroke surgery.
4. Advanced Maternal Age Pregnancy and Stroke (AMAPS) Group: Rats experienced a pregnancy at 10 months of age, and at 20 months of age, the animals underwent stroke surgery.

Female virgin rats were paired with males at a ratio of 2:1. Subsequently, the presence of sperm in the vaginal smear was checked every morning, and those females testing positive for sperm were separated and housed individually [13].

After the end of the lactation period, the pups were separated and the mother animals were maintained until they reached the age of 20 months. All animals were euthanized on 24 h following ischemic stroke surgery. Eight animals in each group were decapitated to obtain brain tissue samples for biochemical analysis and brain tissues of the remaining eight animals in each group were used for histopathological evaluation.

### Transient focal cerebral ischemia

Transient middle cerebral artery occlusion (MCAO) was carried out as previously described [14]. The rats (n = 16 rats/group) were anesthetized with Ketamine (100 mg/kg, intraperitoneal, IP) (Bremer Pharma GMBH, Germany) and Xylazine (10 mg/kg, IP) (Alfasan, Netherlands)). The body temperature was maintained at 37.0 ± 0.5 °C with heating pads, and rectal temperature was monitored throughout the surgical procedure.

After making a midline neck incision, the right common carotid artery (CCA) and external carotid artery (ECA) were exposed and meticulously separated from the vagus nerve. To induce middle cerebral artery occlusion (MCAO), a silicone-coated nylon monofilament was inserted through the external carotid artery (ECA) into the internal carotid artery and further into the circle of Willis until a slight increase in resistance was felt [14].

Afterward, the filament was anchored by tying a silk suture around the external carotid artery (ECA). Blood flow in the middle cerebral artery (MCA) territory was evaluated using laser-Doppler flowmetry (LDF) (moorVMS-LDF1-HP, Moor Instruments Ltd., Oxford, England). The successful induction of middle cerebral artery occlusion (MCAO) was verified by observing a consistent decrease in the laser-Doppler flowmetry (LDF) signal to below 25 % of the baseline measured before the ischemic event. After a duration of ninety minutes, the filament was withdrawn to permit reperfusion of the brain tissue. Subsequently, the rats were given time to recuperate and were monitored in accordance with the experimental protocol. Sham-operated rats underwent surgical preparation of the external carotid artery (ECA) for filament insertion, but the filament was not inserted. [14].

### Assessment of neurological deficits

Neurological deficits (n = 14–16 rats/group) were assessed 24 h after MCAO. The neurological status of each rat was evaluated using a scoring system described in prior research [15]. The neurological scoring system for rats is defined as follows: A score of 0 indicates no neurological deficits observed, a score of 1 indicates that the rat cannot fully straighten its left front paw, a score of 2 indicates leftward rotation while moving, a score of 3 indicates leftward inclination during locomotion, and a score of 4 indicates the rat is incapable of walking on its own and displays symptoms of convulsions, drowsiness, and unconsciousness.

### Measurement of infarction volume

After evaluating neurological deficits, the animals were anesthetized (n = 5 rats/group), and their brains were removed from the skull. Then, the brains were sliced using a rat brain matrix set to a thickness of 2 mm. Subsequently, the brain sections were submerged in a 2 % Triphenyl Tetrazolium Chloride (TTC) solution following established procedures [13]. Images of the brain slices were captured using a scanner manufactured by Hewlett-Packard, USA, and analyzed using ImageJ software (NIH) to quantify the size of the ischemic area.

The following formula was used to calculate the ischemic area volume [13], [16]:

**Volume of the ischemic region**: (volume of the contralateral hemisphere − the volume of the nonischemic region of the ipsilateral hemisphere)/volume of contralateral hemisphere × 100.

### Determination of Blood-Brain barrier permeability

The permeability of the Blood-Brain Barrier was assessed by quantifying the concentration of Evans Blue (EB) dye outside the blood vessels using a spectrophotometer [17]. EB is utilized as an indicator of a significant rise in permeability [17]. Twenty-four hours post MCAO, four rats from each group were anesthetized with ketamine (100 mg/kg) and xylazine (10 mg/kg), followed by the administration of 20 mg/kg Evans Blue dye 2 % (1 ml/kg) through the tail vein. One hour after EB injection, the saline solution was injected through the left ventricle of the heart until the clear solution exited the right atrium. One hour after EB injection, saline was infused through the left ventricle of the heart until the clear solution exited the right atrium to remove intravascular EB. Then the brain tissue was removed from the skull and homogenized in phosphate buffered saline and at that time centrifuged. The supernatant was diluted in 500 μl of trichloroacetic acid overnight at 4 °^C^. After centrifugation at 21,000 g for 30 mins, EB was measured using a spectrophotometer at 620 nm absorption [17]. Results are expressed as the mg/mg brain tissue.

### Real-time quantitative PCR (RT-qPCR)

In this research project, RT-qPCR (Reverse Transcription Quantitative Polymerase Chain Reaction) was utilized to assess the expression levels of VEGF, MMP9, and occludin mRNA (n = 5–6 rats/group). Total RNA was isolated from the ischemic cerebral cortex hemisphere using TRIzol reagent (Invitrogen, Shanghai, China). The isolated RNA underwent reverse transcription to create complementary DNA (cDNA) using the cDNA Reverse Transcription kit (Qiagen, USA) following the provided instructions. Amplification of the cDNAs was carried out with the miScript SYBR Green PCR Kit (Qiagen) as per the manufacturer's recommendations.

The primer sequences for VEGF [18] were as follows: the forward primer was 5′-ACGGGCCTCTGAAACCATGAA-3′ and the reverse primer was 5′-TTTCTGCTCCCCTTCTGTCGT-3′. For MMP9 [18], the forward primer was 5′‑GCCGGGAACGTATCTGGAAA‑3′ and the reverse primer was 5′‑GGTTGTGGAAACTCACACGC‑3′. The primer sequences for Occludin [15] were: the forward primer 5′-TGTGTTCCCCCAGGTAGACT-3′ and the reverse primer 5′-GGTCACACAGTGACACTCCA-3′. Additionally, the internal reference gene β-actin [19] was included, with the forward primer sequence being 5′- GGAGATTACTGCCCTGGCTCCTA-3′ and the reverse primer sequence being 5′- GACTCATCGTACTCCTGCTTGCTG-3′.

### Biochemical evaluations

Biochemical evaluations included measuring the levels of the Tumor necrosis factor alpha (TNF-α) and total oxidant capacity (TOC) levels, in the 24 h following cerebral ischemia. TNF-α was measured using an ELISA kit from R&D Systems (USA), and TOC levels were assessed using a Kiazist kit (Iran), following the manufacturer’s protocols.

Rats were anesthetized and sacrificed 24 h after cerebral ischemia, following a neurological assessment. Samples of ischemic brain tissue (40 mg) were obtained and placed in a cold buffer (0.01 M phosphate-buffered saline, pH 7.4, containing a protease inhibitor cocktail [Roche]). The samples were then homogenized and centrifuged at 12,000 g for 15 min at 4 °C, and the supernatants were collected.

After preparing the samples, the following steps were undertaken. All solutions were brought to room temperature.

### Total oxidant capacity (TOC) measurement

Based on manufacturer’s protocol, the working solution was prepared in a tube by mixing 200 μl of TOS Buffer with 2 μl of Developer solution for each sample. In a separate tube, 10 μl of the standard vial was mixed with 10 mL of deionized water to create a 10 mM standard. Then, 1000 μl of deionized water was added to a microtube, from which 5 μl was discarded; instead, 5 μl of the 10 mM solution was added to make a final concentration of 50 µM H2O2. Next, 500 μl of deionized water was added to five microtubes. From the 50 µM concentration, 500 μl was added to the first microtube and mixed well to prepare a serial dilution. Fifty microliters of each sample or standard was added to each well, with PBS buffer used as a blank. Two hundred microliters of the working solution was added to each well. The wells were incubated at room temperature for 15 min, after which absorbance was measured at 560 nm.

### Tumor necrosis factor-α measurement

Based on manufacturer’s protocol, fifty microliters of all samples and standards were added to the appropriate wells. Subsequently, 100 µl of Conjugate was added to each well. The plate was sealed and incubated for two hours at room temperature. After incubation, the contents were aspirated and washed five times. One hundred microliters of Substrate Solution was added to each well and incubated for 30 min in the dark on a plate shaker. Finally, 100 µl of Stop Solution was added to each well, and the optical density (OD) at 450 nm was recorded.

### Statistical analysis

The analysis of the data was carried out with GraphPad Prism Ver. 10 software. A one-way ANOVA was used for statistical analysis, followed by Bonferroni tests. Mann-Whitney *U* test was used for neurological deficit score. The results are shown as mean values with standard error of the mean (SEM). A significance level of P<0.05 was deemed to indicate a significant distinction.

## Results

### Mortality

Post-MCAO surgery, mortality rates were not significantly different among the sham, ISO, YPS, and AMAPS groups (p = 0.5127). While the sham group experienced no mortality, the ISO group lost 2 animals, the YPS group lost 1 animal, and the AMAPS group lost 2 animals. Data from dead animals were excluded from the analysis.

## Neurological deficit scores and tissue damage after stroke induction in rats

**Fig. 2:**
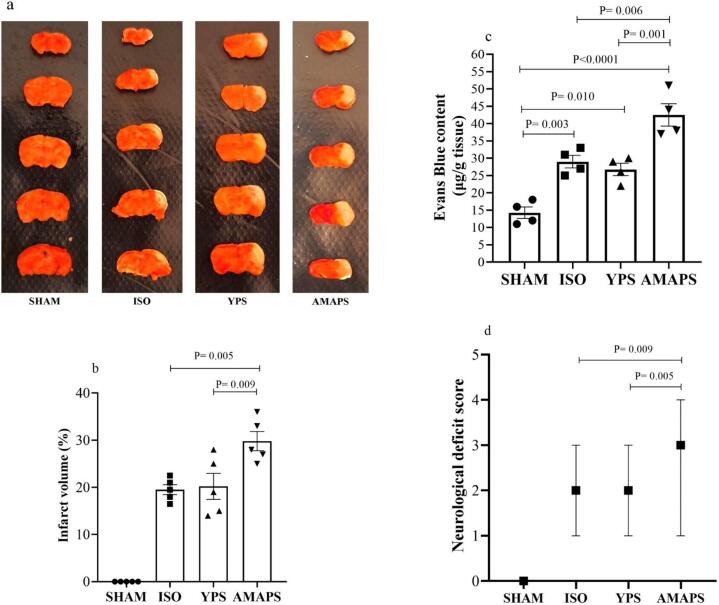
Displays the (a-b) infarct volume (n = 5 rats/group), (c) brain Evans blue content (n = 4 rats/group) and (d) scores for neurological deficits (n = 14–16 rats/group) in the SHAM, ISO, YPS, and AMAPS groups. (For interpretation of the references to colour in this figure legend, the reader is referred to the web version of this article.)

The examination of brain infarct volume indicated that the infarct area in the AMAPS group was notably larger than that in the YPS (F (3,16) = 48.81, p = 0.009) and ISO (F (3,16) = 48.81, p = 0.005) groups, as depicted in Fig. 2a-b.

To evaluate blood–brain barrier (BBB) integrity, the parenchymal Evans blue (EB) content was quantified. The EB content in the AMAPS group was significantly higher than that in the YPS (F (3,12) = 27.18, p = 0.001), ISO (F (3,12) = 27.18, p = 0.006), and SHAM (F (3,12) = 27.18, p < 0.0001) groups, as illustrated in Fig. 2c. The SHAM group exhibited significantly lower EB content compared to both the ISO group (F (3,12) = 27.18, p = 0.003) and the YPS group (F (3,12) = 27.18, p = 0.010).

As demonstrated in Fig. 2d, the assessment of neurological deficit scores revealed a significantly higher score in the AMAPS group compared to both the YPS (p = 0.005) and ISO (p = 0.009) groups, indicating a more severe neurological condition.

## VEGF, occludin and MMP-9 gene expressions in ischemic brain tissue

**Fig. 3:**
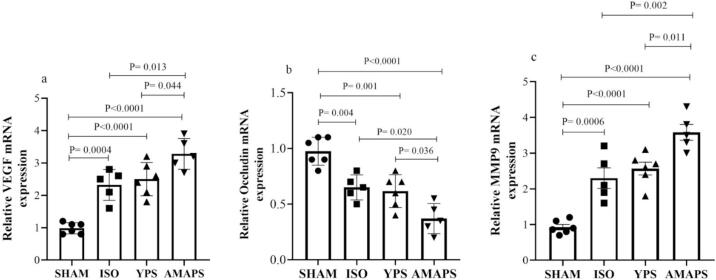
Depicts the mRNA expression levels of (a) VEGF (n = 5–6 rats/group), (b) Occludin (n = 5–6 rats/group), and (c) MMP9 (n = 5–6 rats/group) in the brain ischemic tissue across the SHAM, ISO, YPS, and AMAPS groups.

In Fig. 3a, the relative VEGF mRNA expression level in the AMAPS group showed significantly higher levels compared to both the YPS group (F (3,18) = 27.98, p = 0.044) and the ISO group (F (3,18) = 27.98, p = 0.013). The SHAM group exhibited significantly lower VEGF mRNA expression levels than the ISO (F (3,18) = 27.98, p = 0.0004), YPS (F (3, 18) = 27.98, p < 0.0001), and AMAPS (F (3, 18) = 27.98, p < 0.0001) groups. Furthermore, as depicted in Fig. 3b, the AMAPS group had a notably reduced relative occludin mRNA expression in contrast to the YPS (F (3, 18) = 19.87, p = 0.036), ISO (F (3, 18) = 19.87, p = 0.020) groups. The SHAM group displayed significantly higher relative occludin mRNA expression levels compared to the ISO (F (3, 18) = 19.87, p = 0.004), YPS (F (3, 18) = 19.87, p = 0.001) and AMAPS (F (3, 18) = 19.87, p < 0.0001) groups. Lastly, Fig. 3c highlights that the relative MMP-9 mRNA expression in the AMAPS group was significantly elevated in comparison to the YPS (F (3, 18) = 31.48, p = 0.011) and ISO (F (3, 18) = 31.48, p = 0.002) groups. Conversely, the SHAM group had notably lower relative MMP-9 mRNA expression levels relative to the ISO (F (3, 18) = 31.48, p = 0.0006), YPS (F (3, 18) = 31.48, p < 0.0001), and AMAPS (F (3, 18) = 31.48, p < 0.0001) groups.

### Levels of TNF-α and total oxidant capacity in ischemic tissue

**Fig. 4:**
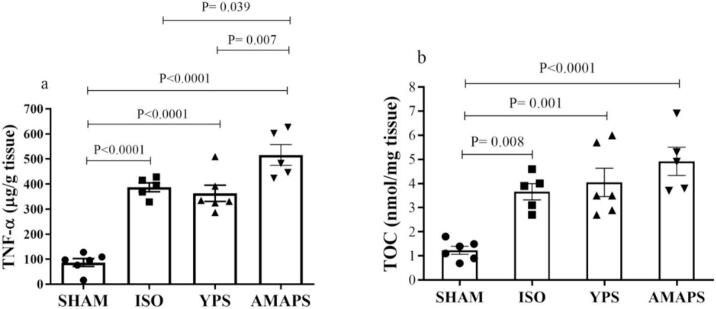
Presents the levels of TNF-α (n = 5–6 rats/group) and TOC (n = 5–6 rats/group) in ischemic tissue across the SHAM, ISO, YPS, and AMAPS groups.

As depicted in Fig. 4a, the findings reveal a significant increase in TNF-α levels in the AMAPS group compared to the YPS (F (3, 18) = 41.49, p = 0.007), ISO (F (3, 18) = 41.49, p = 0.039) groups. Furthermore, TNF-α levels in the SHAM group were significantly lower than ISO (F (3, 18) = 41.49, p < 0.0001), YPS (F (3, 18) = 41.49, p < 0.0001) and AMAPS (F (3, 18) = 41.49, p < 0.0001) groups.

Regarding total oxidant capacity (TOC) levels (Fig. 4b), the data showed that the AMAPS group had higher TOC levels compared to the ISO and YPS groups, although this difference was not statistically significant. Furthermore, the mean TOC level in the SHAM group was significantly lower than ISO (F (3, 18) = 12.65, p = 0.008), YPS (F (3, 18) = 12.65, p = 0.001) and AMAPS (F (3, 18) = 12.65, p < 0.0001) groups.

## Discussion

The findings of this study align with existing clinical evidence that suggests advanced maternal age (AMA) is associated with increased cardiovascular risks and adverse outcomes, including ischemic stroke. Previous studies have demonstrated that AMA is linked to heightened inflammatory responses, oxidative stress, and vascular dysfunction, which are critical factors in the pathogenesis of ischemic stroke [3], [9], [20].

In our study, we observed significantly elevated levels of MMP-9 expression and reduced levels of occludin in AMA rats, which are consistent with clinical observations of increased blood–brain barrier (BBB) permeability and vascular damage in older individuals [21]. Additionally, the higher levels of TNF-alpha and total oxidant capacity in AMA rats further support the notion that inflammatory and oxidative mechanisms are exacerbated in this population, contributing to greater ischemic damage.

Comparing our findings with clinical studies, it is evident that the physiological changes associated with AMA [3], [9], [20], such as increased oxidative stress and inflammation, mirror those observed in aged human populations. This similarity underscores the relevance of our animal model in understanding the impact of AMA on ischemic stroke outcomes.

The implications of our study are significant, as they highlight the need for targeted interventions to mitigate the heightened risk of ischemic stroke in women of advanced maternal age. Clinicians should be aware of the increased vulnerability in this population and consider proactive measures, such as monitoring and managing cardiovascular risk factors, to improve maternal and fetal outcomes. This study provides insights into the complex interplay between pregnancy-related physiological changes, aging, and ischemic stroke. The observed similarities between our findings and clinical evidence emphasize the importance of addressing the unique challenges faced by women of advanced maternal age to reduce the burden of ischemic stroke.

AMA is associated with an increased risk of stroke in later life [22], [23]. This risk is multifaceted, involving age-related vascular changes, pregnancy-induced hemodynamic stress, and a higher prevalence of comorbidities such as hypertension and diabetes [22], [23]. The physiological demands of pregnancy at an older age can exacerbate these factors, potentially leading to a higher incidence of ischemic stroke [22], [23].

In our study observed increase in infarct volume, BBB permeability, and neurological deficits in AMA rats compared to their younger counterparts aligns with emerging data on the impact of maternal age on cerebrovascular events [22], [23].

The heightened susceptibility to stroke in AMA rats can be ascribed to age-related physiological alterations. Aging is linked to reduced vascular flexibility and heightened pro-inflammatory biomarkers [24], potentially amplifying the impact of pregnancy on the cerebrovascular system. Pregnancy induces notable metabolic and hemodynamic shifts in a woman's body to facilitate fetal development [25]. Inadequate adaptation to these changes could precipitate gestational hypertension complications (hypertension, pre-eclampsia, or eclampsia), gestational diabetes, and premature delivery [25]. Contrary to previous assumptions, these complications are not confined to pregnancy, potentially resulting in lasting vascular and metabolic impairment [25]. Additionally, a direct association exists between these conditions and an elevated risk of future cardiovascular ailments such as stroke, hypertension, ischemic heart disease, heart failure and diabetes [25]. In addition, in older age, the post-pregnancy recovery processes may be less effective, leading to a slower and less robust return to pre-pregnancy physiological states [26], [27].

Giller et al. (2020) proposed similarities between aging pathology and pregnancy; however, while pregnant women exhibit recovery, aging represents enduring decline [26]. The physiological and cellular deterioration observed during pregnancy mirrors mechanisms linked to aging [26].

Interestingly, in a recent study, Pham et al. (2024) discovered that pregnancy temporarily accelerates biological aging, meaning that pregnancy increases biological age but there is a noticeable recovery in the postpartum period [27].

Aging displays a continual decline lacking recovery, contrary to pregnancy which showcases extraordinary rejuvenation post-stress, restoring damage to pre-pregnancy levels postpartum, [26], [27]. It is plausible that pregnancy at an advanced maternal age (AMA) accelerates aging, amplifying the impact of age-related diseases. Thus, in our study, the increased infarcted area volume, cerebral edema, and neurological deficits in AMA group animals compared to young-aged pregnancies may stem from accentuated aging processes in the AMA group.

Our study revealed that AMA, when compared to pregnancy at a young age in rats, is associated with increased levels of vascular endothelial growth factor (VEGF) and matrix metalloproteinase-9 (MMP9) expressions, along with reduced expression of occludin, leading to increased cerebral edema in ischemic brain tissue post-stroke at old age. These findings suggest that AMA may exacerbate the pathophysiological processes following an ischemic stroke at old age.

In our study, while the rise in VEGF levels in the AMA group did not reach statistical significance compared to the other groups, it was higher. VEGF is a key regulator of angiogenesis and vascular permeability. Elevated VEGF levels in AMA rats could indicate a compensatory response to ischemic injury, aimed at restoring cerebral blood flow [28]. However, excessive VEGF can also increase blood–brain barrier (BBB) permeability, leading to vasogenic edema [18].

The relationship between VEGF and AMA is a significant area of interest in reproductive biology and obstetrics [22]. VEGF is a critical signaling protein involved in both vasculogenesis and angiogenesis, playing a vital role in the development of the placenta and maintenance of pregnancy [22], [29]. In the context of AMA, which is generally considered to be pregnancy at the age of 35 or older, the regulation of VEGF becomes even more crucial due to the increased risk of adverse obstetrical and perinatal outcomes [29], [30].

Research has indicated that AMA is associated with a higher incidence of pregnancy-induced hypertension, antepartum hemorrhage, cesarean delivery, preterm delivery, low birth weight, perinatal death, and low fifth-minute APGAR scores [30]. These complications can be linked to the altered expression and function of VEGF [29], [31]. As women age, there may be changes in the endothelial function and a decline in the efficiency of angiogenic processes, which could affect the levels and activity of VEGF [29], [31].

Furthermore, the balance between VEGF and its inhibitors is crucial for normal placental development [32]. Disruption in this balance can lead to conditions such as preeclampsia, which is characterized by high blood pressure and often proteinuria [32]. Preeclampsia is more common in AMA pregnancies and has been associated with changes in VEGF expression [29], [31], [32]. The exact mechanisms by which AMA affects VEGF levels and function are not fully understood, but they are likely to involve a combination of genetic, environmental, and physiological factors [33].

Evidence suggests that altered levels of angiogenic growth mediators (eg, VEGF and PlGF) are a common mechanism by which AMA leads to pregnancy complications in pregnancy-related diseases [33]. It seems that modifying the expression of VEGF and PlGF in pregnancies with AMA can help improve some pregnancy complications [33].

MMP9, an enzyme involved in the degradation of the extracellular matrix, is known to contribute to BBB disruption following ischemic events [34]. In our study, its upregulation in AMA rats could further facilitate the breakdown of BBB integrity, exacerbating cerebral edema [34].

MMP9 is an enzyme that plays a significant role in the aging process [34]. Its activity is a double-edged sword; while necessary for normal physiological functions, excessive MMP9 activity can contribute to age-related diseases [34]. As we age, the balance between the synthesis and degradation of extracellular matrix components can be disrupted, that MMP9 activity has role in this process [35].

Studies have demonstrated that Matrix Metalloproteinases (MMPs) play a crucial role in the aging process of various tissues [35]. Excessive activity of MMP9 can result in the degradation of collagen and elastin, contributing to visible signs of aging and decreased tissue efficiency [35].

Furthermore, MMPs are implicated in the progression of age-related diseases, including neuropathologies, cardiomyopathies, inflammatory conditions, fibrosis, and cancer [35]. Dysregulation of MMP function can exacerbate tissue damage and inflammation, thereby influencing the development and advancement of these age-related disorders [35].

Recent studies have illuminated the intricate relationship between pregnancy and biological aging [26], [27]. Pregnancy itself can lead to biological changes that resemble aspects of aging [26], [27].

Hormonal shifts, metabolic alterations, and cellular stress during pregnancy contribute to this phenomenon [26], [27]. Perhaps, in cases where pregnancy takes place at an older age, the convergence of maternal aging and pregnancy-associated factors could potentially magnify the expression of MMP9. This increased MMP9 response might be particularly significant following a stroke in elderly individuals. The interaction between aging induced by pregnancy and the inherent aging process could synergistically impact MMP9 expression. Subsequently, this heightened MMP9 level post-stroke may have implications for outcomes such as infarct volume and brain edema.

Our study unveils a notable disparity in occludin expression levels between rats that underwent pregnancy at an advanced age and those with pregnancies at a younger age. Following a stroke, both groups experienced alterations in occludin expression, with a more pronounced effect observed in rats with pregnancies at an older age. The decline in occludin levels post-stroke could pose a risk to blood–brain barrier (BBB) integrity, potentially worsening brain damage. The heightened infarct volumes detected in rats with pregnancies at an advanced age underscore the seriousness of brain tissue damage. Additionally, increased edema following the stroke contributes further to unfavorable outcomes.

While our findings indicate increased BBB permeability in AMA rats, it is crucial to interpret these results with prudence. The observed increase in Evans blue extravasation is likely attributable to the larger infarct size in AMA rats, rather than a direct effect of AMA on the brain vasculature. This distinction is important for accurately understanding the underlying mechanisms contributing to BBB disruption in the context of advanced maternal age and ischemic stroke.

Based on these findings, it can be concluded that pregnancy at an older age may exacerbate the inflammatory response and oxidative stress in the brain following a stroke. This suggests that advanced maternal age may be a risk factor for increased neuroinflammation and oxidative damage in the brain after a stroke. Additionally, the higher levels of VEGF, MMP9, infarct volume, and edema in older pregnant mice further support the idea that pregnancy at an older age may have detrimental effects on stroke outcomes. Overall, these results highlight the importance of considering maternal age as a potential factor influencing post-stroke outcomes and suggest that further research is needed to better understand the mechanisms underlying these effects.

## Conclusion

In conclusion, our study highlights the potential impact of Advanced Maternal Age (AMA) on cerebral vulnerability post-ischemic stroke. The findings suggest that pregnancies occurring at the age of 35 or above may predispose the brain to greater damage in the event of an ischemic insult later in life, compared to pregnancies at a younger age. The heightened susceptibility to stroke in AMA rats can be attributed to age-related physiological alterations, pregnancy-induced hemodynamic stress, and increased risk of comorbidities such as hypertension and diabetes. Additionally, our study revealed alterations in key biomarkers associated with angiogenesis and vascular permeability, indicating potential mechanisms underlying the increased cerebral edema observed in AMA rats post-stroke. These findings underscore the importance of considering maternal age as a factor in assessing stroke risk and developing targeted interventions for older pregnant individuals to mitigate potential cerebrovascular complications later in life. Further research is warranted to elucidate the underlying mechanisms and explore potential therapeutic strategies for this vulnerable population.

## Ethics approval statement

This study was approved by Ethics Committee of Zahedan University of Medical Sciences (ethical code: IR.ZAUMS.REC.1393. 6852).

## Author contributions

Hamed Fanaei and Samira Khayat designed the study. Hamed Fanaei carried out the experiments. Hamed Fanaei and Samira Khayat analysed the data. Hamed Fanaei wrote the manuscript.

## Funding Sources

Financial support for the study was conducted by the Office of Vice-Chancellor for Research and Information Technology of 10.13039/501100004847Zahedan University of Medical Sciences (Grant No. 6852).

## CRediT authorship contribution statement

**Samira Khayat:** Investigation, Conceptualization. **Hamed Fanaei:** Supervision, Resources, Project administration, Methodology, Investigation, Funding acquisition, Formal analysis, Data curation, Conceptualization.

## Declaration of competing interest

The authors declare that they have no known competing financial interests or personal relationships that could have appeared to influence the work reported in this paper.
